# Influence of Perceived Racial Discrimination on the Health of Immigrant Children in Canada

**DOI:** 10.1007/s12134-018-0539-3

**Published:** 2018-02-24

**Authors:** M. Anne George, Cherylynn Bassani

**Affiliations:** 1 Department of Pediatrics, University of British Columbia, Vancouver, British Columbia Canada; 2 BC Children’s Hospital Research Institute, Vancouver, British Columbia Canada; 3 School of Population & Public Health, University of British Columbia, Vancouver, British Columbia Canada; 4 Sociology, Kwantlen Polytechnic University, Surrey, British Columbia Canada

**Keywords:** Immigrant children, Health, Ethnicity, Racial discrimination, Canada

## Abstract

Racial discrimination and racism are recognized as determinants of health for adults. Less is understood regarding the influence of discrimination targeted towards parents, the family, or the cultural and children’s health. Data from the New Canadian Children and Youth Study (NCCYS) are used in this paper. The NCCYS is a national, longitudinal study of children whose families settled in urban centers of Canada. We analyzed data from individuals who settled in the metropolitan Vancouver area from six ethnic communities: Mainland China, Hong Kong, the Philippines, Iran, Afghanistan, and the Punjab who were interviewed at two times, approximately 2 years apart. Data were collected on perceived parental, family, and cultural discrimination. Our dependent variable was parent-reported child health status. Over time, perceived parental discrimination and perceived family discrimination decreased; and both forms of discrimination had a positive effect on child health. In contrast, perceived cultural discrimination increased over time and had a negative effect on child health at both times. Different forms of discrimination have different effects on child health. Racial discrimination is complex. Its influence on either increasing family cohesion, and thereby leading to improved health, or increasing stress, thereby leading to poorer health needs to be explored further.

## Introduction

Racial discrimination and racism are recognized as determinants of health in adult populations (Harris et al. 2015; Paradies et al. 2015; Priest et al. 2013; Pascoe and Smart Richman 2009; Krieger 2000). Limited research has examined this relationship in child populations (Paradies et al. 2015; Bécares et al. 2015).

Racial discrimination is defined as “any distinction, exclusion, restriction or preference based on race, colour, descent, or national or ethnic origin” (United Nations 1969). Racism has been defined in various ways, but generally is considered to be the action or attitude that subordinates individuals or groups of people, through a power relationship, ranking some groups as culturally superior to others (Williams and Mohammed 2009; Jones 1997). It emanates from an organized system with dominant ranking of one or more groups, and leads to restriction of goods or resources to groups allocated as inferior (Williams and Mohammed 2009; Jones 1997). Cultural racism is a fundamental component of racism (Jones 1997) and can lead to racial discrimination (Williams and Mohammed 2009). The United Nations on the Convention of the Rights of the Child states that children must be protected from all forms of discrimination (United Nations 1969).

In adult populations, suggested mechanisms linking racism and poor health outcomes are the following: restricted access to resources or increased exposure to risk factors, negative patho-psychological responses, physiological stress response (allostatic load), reduced uptake of healthy behaviors, or increased uptake of unhealthy practices (Paradies et al. 2015; Priest et al. 2013; Williams and Mohammed 2009; Pascoe and Smart Richman 2009; Brondolo et al. 2009; Williams et al. 2008; Miller and Kaiser 2001; Clark et al. 1999). Thus, both direct and indirect pathways may influence health outcomes (Bécares et al. 2015). Longitudinal studies have found that discrimination directly precedes negative health outcomes (Bécares et al. 2015; Paradies et al. 2015; Pascoe and Smart Richman 2009; Williams and Mohammed 2009; Gee and Walsemann 2009). Studies have found that coping style, social support, ethnic identity, and personality may moderate the association between perceived discrimination and mental health outcomes or unhealthy behaviors (Brondolo et al. 2009; Pascoe and Smart Richman 2009; Noh and Kaspar 2003; Noh et al. 1999).

Causal pathways have not been determined because of the nonexperimental design of studies (Paradies et al. 2015; Brondolo et al. 2009; Pascoe and Smart Richman 2009), although longitudinal studies are suggestive of causation because discrimination precedes the negative health outcomes (Paradies et al. 2015; Pascoe and Smart Richman 2009; Williams and Mohammed 2009; Gee and Walsemann 2009).

In spite of the extensive literature linking racial discrimination and health outcomes, limited research has been conducted on the effect of racial discrimination on the health of immigrant children, though an emerging literature is aimed at understanding its effects (Bécares et al. 2015; Tran 2014; Oxman-Martinez and Choi 2014; Priest et al. 2013; Halim et al. 2013; Beiser et al. 2012; George et al. 2012; Oxman-Martinez et al. 2012; Pachter and Coll 2009; Finch et al. 2000; Fisher et al. 2000). Associations between perceived racism and mental health, socioemotional development, or behavioral outcomes have been found in the child and youth literature (Bécares et al. 2015; Tran 2014; Priest et al. 2013; George et al. 2012; Pachter and Coll 2009), with recent studies also examining academic performance (Oxman-Martinez and Choi 2014; Oxman-Martinez et al. 2012). Halim et al. (2013) found perceived language-based discrimination to be associated with more frequent visits to the doctor for child-related sickness, with ethnic group attachment being a mitigating factor.

Racial discrimination experienced by parents appears to influence the child’s health directly or indirectly as a function of family stress and attitudes, or by parents becoming less able to provide a caring environment for their children (Bécares et al. 2015; Tran 2014; Kelly et al. 2013). It has been found that racial discrimination is associated with the mother’s mental health, which in turn influences the mental health or socioemotional development of the child (Tran 2014; Bécares et al. 2015; Kelly et al. 2013).

The extant child literature is limited by a lack of cohort or longitudinal studies and, similar to adult based literature, by the lack of diversity in samples (Pachter and Coll 2009; Priest et al. 2013; Noh et al. 2007). Most studies have been conducted in the United States (USA) within the large African-American, Latino, or Asian populations (Priest et al. 2013; Pachter and Coll 2009). Pachter and Coll (2009) found in their review of the literature that 70% of 40 articles were conducted on African-American samples, while Priest et al. (2013) found only three refugees samples and 46 immigrant samples in the 121 studies that they reviewed. Most of the latter singled out specific immigrant populations. Among the suggestions for future research emanating from these reviews were having more ethnically diverse minority samples (Pachter and Coll 2009) and the use of longitudinal data (Priest et al. 2013). Again, in accordance with adult literature, most studies focused on mental health outcomes (Williams and Mohammed 2009).

Our New Canadian Child and Youth Study (NCCYS) contributes to the literature in a number of ways since, with the exception of the UK Millennium Cohort Study (Bécares et al. 2015; Kelly et al. 2013), very few studies on racial discrimination have been conducted outside the dominant populations of African-American and Latinos in the USA. From the NCCYS data, we examined the long-term influence of parent’s racism experiences. Few other studies of children measure general health rather than mental or emotional health or specific illnesses. Our study focuses on immigrant and refugee children settling in Canada from six regions or countries: Afghanistan, Iran, Hong Kong, Mainland China, the Philippines, and the Punjab region of India. In addition, we use a longitudinal panel design which means that we can model causality. As well, we study three measures of parent-perceived racial discrimination: that experienced by the individual parent, the family, and the culture. Further, in contrast to most studies that focus on specific mental (e.g., depression) or physical conditions (e.g., obesity or health behaviors), our study examines children’s general health.

## Methods

We report findings from the two data collection points of the NCCYS, a national, longitudinal study of children whose families emigrated to six urban centers in Canada (George and Bassani 2013, 2015; George et al. 2012; Bassani and George 2012; Beiser et al. 2010, 2011; Oxman-Martinez and Choi 2014; Oxman-Martinez et al. 2012). Our analysis includes data from individuals who settled in the metropolitan Vancouver area from six ethnic groups: Mainland China, Hong Kong, the Philippines, Iran, Afghanistan, and the Punjab who were interviewed at both Times 1 and 2 of the NCCYS.

Structured questionnaires were used to collect data from each family in their homes. Information was recorded concerning demography, physical health and mental health, family, migration experience, social supports, work history, and child experiences. The primary care giver (usually the mother) (PK) was interviewed about their families and about their children.

### Sample

Sample inclusion factors were the following: immigration from any of the six specific countries or regions, had lived in Canada for 10 or fewer years, were fluent in the dominate language of their country of origin, and had at least one child in either of the two developmentally formative age groups (4–6 years or 11–13 years). Participants from these specific countries and dominant language groups were selected because they represented the current ethnic makeup of these Canadian cities. For example, Punjabi-speaking people from India made up the largest community emigrating from India to the metropolitan Vancouver area so this group was included in the study.

Quota sampling was employed to ensure an equal sample size from each immigrant group. During initial recruitment, for each ethnic community, 180 children were included: 90 children from each age cohort.

Because complete lists of immigrant populations do not exist in Canada, various sampling techniques were used with an aim to provide sample heterogeneity. Complementing quota sampling, snowball and purposive sampling were used. Four of the ten school boards in the region sent letters addressed to the parents of children who appeared to fit the eligibility criteria. Families responded directly to their school boards, permitting us to follow up to ensure eligibility. The study was also widely promoted through ethnic media and at ethno-cultural or religious events and community centers, parenting programs, language schools, and malls. As well, through snowball sampling, study participants referred others fitting the eligibility criteria.

More than one child per family could be included in the study provided they met the cohort requirements. To maintain the statistical assumption of independence of participants, the present analysis includes only one randomly drawn child per family. At Time 1, our Vancouver sample size was 1081. This was reduced to 855 at Time 2 due to attrition. After deleting siblings, the sample size was reduced 627.

### Measures

#### Dependent Variable

One dependent variable was assessed, parent rated health status of the child. Self-rated health (or in this case, rating by the parent) has become increasingly popular as a global measure of health, and has methodologically been found to be a reliable and valid predictor of general health or specific illnesses across communities, including different cultures and adult age groups, and educational backgrounds (Huisman et al. 2007; Quesnel-Vallee 2007; Benyamini and Idler 1999; Idler and Benyamini 1997). Parents were asked: “In general, would you say your son’s/daughter’s health is: excellent, very good, good, fair or poor.” This categorical question was dummy coded, comparing those with excellent health to those who do not have excellent health. Dummy coding this variable has been found to be both useful and valid depending on the research question (Manor et al. 2000) or the quality of the sample—considering issues such as the size of age range (Finnäs et al. 2008). Some researchers choose to include two categories (combining very good and excellent) compared to other categories (e.g., Setia et al. 2011). Based on past research (George et al. 2012; Manor et al. 2000), the qualitative nature of the variable and the variable’s distribution were elected to contrast excellent vs nonexcellent health.

#### Independent Variables

Following our previous measures of perceived discrimination (George et al. 2012) that were based on Karlsen and Nazroo’s (2002) contextualized ideology of racism, three levels of racial discrimination were measured: individual (parent), family, and cultural. For parent-perceived discrimination, parents were asked how often they have felt stress in any of four stated incidences (see Appendix). Response categories were never, sometimes, often, very often, or not applicable, with the latter coded as a system missing response. As displayed in Table 1, the parent discrimination variable ranged from 0 (no discrimination) to 12 (frequent discrimination), with a mean of 1.70 at Time 1 (T1) and 0.81 at Time 2 (T2).

**Table 1:** Descriptive statistics by Time, NCCYS, 2002 and 2006, *n* = 627

	Mean (SD)		Minimum-maximum values	
	Time 1 (627)	Time 2 (627)	Time 1 (627)	Time 2 (627)
PK discrimination	1.70 (1.84)*	0.81 (1.26)	0–9.00	0–8.00
Family discrimination	0.47 (0.83)*	0.24 (0.70)	0–5.00	0–6.00
Culture discrimination	8.74 (3.51)*	9.73 (4.48)	0–22.00	0–24.00
Income	5.60 (2.99)* $24,000 to $24,999	9.20 (4.44) $45,000 to $54,999	0–12.00 $0.00 to $80,000 +	0–12.00 $0.00 to $80,000 +
	Percentage			
	Time 1		Time 2	
PK has University	42.36%		61.10%	
Child in excellent health	30.67% +		41.32%	
Sex Female Male	ᅟ 49.5% (310)* 50.5% (318)			
Mean years in Canada	7.86 years			
Actual year range	2 to 19 years			

*T test statistically significant, *p* = 0.001

+ Chi square statistically significant, *p* = 0.001

Perceived family racial discrimination was measured by compiling the responses of parents’ self-report of anyone in the family experiencing any of six specific incidences in the past 12 months (see Appendix). With dichotomous responses (either positive or negative), the family discrimination scale ranges from 0 (no discrimination) to 6 (highly discriminated) and has a mean of 0.47 (T1) and 0.24 (T2).

Perceived cultural discrimination was measured by compiling responses of parents’ self-report to six items that asked how strongly parents agreed with statements about general discriminatory practices; for example, if Canadians tend to look down on their cultural group or treat negatively in stores. Six responses ranged from strongly disagree to strongly agree. Response category “not sure” was recoded to the missing response since it suggests that the respondent does not know the answer to the question. As indicated in Table 1, this scale ranges from 0 (no perceived discrimination) to 18 (perceived discrimination) and has a mean of 8.74 (T1) and 9.73 (T2).

#### Control Variables

Four variables are included as controls: household income (at Times 1 and 2), parent’s education (at Times 1 and 2), child’s sex, and year of parent’s arrival in Canada. Each of these variables have been found in the literature to be vital in understanding immigrant children’s health in Canada (George and Bassani 2013, 2015; George et al. 2012; Beiser et al. 2011, 2012, 2014; Tran 2014). Household income was measured with a closed-ended 12-item response variable, ranging from $0.00 to over $80,000 per year. While ordinal, we classified income as continuous due to the large number of response categories. Missing cases were coded to the mean.

Parent’s education was based on highest level of education of the respondent, typically the mother, at Times 1 and 2. The categorical variable was dummy coded to either having a university degree or not having a university degree. Education was dummy coded so to contrast the influence the effect of higher parental education with lower.

Sex of child and parent’s arrival in Canada are static variables. Year of arrival in Canada was a continuous variable.

### Statistical Analysis

Descriptive and logistic regression analyses were conducted. We report means and percentages for continuous and categorical variables, respectively, for Times 1 and 2. *T* tests were conducted to report statistical variance between Times 1 and 2 for the continuous variables and chi square was conducted to report variance over time for categorical variables. A criterion *p* level of 0.001 was established.

Hierarchical linear modeling (HLM) was used to assess the effect, over time that perceived ethnic discrimination had on the likelihood of a child being in excellent health. A two-level hierarchical model was developed to account for multiple observations that are nested within a single person (35). Level one includes “interview” level data: time variable (either Time 1 or Time 2) and all static variables that do not fluctuate over time. Level 2 included non-static participant variables: parent report excellent health, the three discrimination measures, and two control variables, income and education.

Dummy coded variables were left uncentered, while continuous variables were centered on the sample mean. This paper reports two logistic regression models: odds ratios and confidence intervals.

The Bernoulli distribution (logistic regression) was used. The odds ratio of the occurrence of excellent health is reported. Given our cells sizes and that our dependent variable was originally ordinal, we choose binary logistic regression over ordinal logistic regression. 95% confidence intervals (CI) are reported. While specific statistics are provided in Table 2, we avoided reporting specific coefficients, because the sample is non-random. Instead, we report and discuss the trends. SPPS 22.0 and HLM 7.0 software were used.

**Table 2:** Logistic regression+ of the influence of perceived discrimination on parent rated health of child, NCCYS 2002 and 2006, *n* = 627

	Model 1		Model 2	
	OR	CI	OR	CI
Intercept L2	*0.29*	0.22–0.39	*0.39*	0.27–0.56
Level 1 (varying measures)				
Time (T)	*1.72*	1.33–2.23	0.95	0.53–1.70
Parent-perceived discrimination	*1.13*	1.04–1.23	*1.19*	1.07–1.31
Time-by-parent discrimination			0.91	0.77–1.06
Family perceived discrimination	1.09	0.93–1.27	*1.27*	1.03–1.56
Time-by-family discrimination			0.76	0.56–1.04
Culture-perceived discrimination	*0.94*	0.90–0.96	*0.88*	0.83–0.93
Time-by-culture discrimination			*1.10*	1.03–1.18
PK income	1.02	0.98–1.06	1.02	0.99–1.06
PK University educated	*1.62*	1.27–2.08	*1.62*	1.27–2.08
Level 2 (static measures)				
Female	1.15	0.88–1.50	1.16	0.89–1.51
Year of arrival in Canada	0.96	0.92–1.01	0.96	0.92–1.01

+Robust standard errors

### Ethics

The Behavioural Research Ethics Board at the University of British Columbia approved the methods.

## Results

Tables 1 and 2 report the descriptive and logistic regression results.

Based on *t* tests, there is a statistically significant (*p* = 0.001) mean difference between T1 and T2 across all continuous variables. As we can see in Table 1, the proportion of children reported as having excellent health (T1 = 30.67%, T2 = 41.32%) increased.

Both perceived individual (T1 μ = 1.70, T2 μ = 0.81) and family (T1 μ = 0.47, T2 μ = 0.24) discrimination decreased between the two time points; though perceived cultural discrimination (T1 μ = 8.74, T2 μ = 9.73) experienced by the parent increased. As noted by contrasting the range to the respective mean, we found that both perceived individual and family levels discrimination were low. Respondents reported higher levels of cultural discrimination. Between the two time points, culture discrimination increased (T1 μ = 8.74, T2 μ = 9.73).

Table 1 shows that the sample was nearly evenly divided by gender (males 50.6%; females 49.4%). The primary care giver (PK) had lived in Canada for an average of 7.86 year when interviewed, though the amount of time that the PK had lived in Canada ranged from 2 to 19 years. Between the two times, the proportion of parents with a university education increased (T1 = 42.36%, T2 = 61.10%). Family income also increased (T1 μ = $20, 000 to $24, 999), (T2 μ = $45, 000 to $54, 999).

Table 2 reports two logistic regression models. Model 1 reports general equation results of the effect of the three discrimination measures and controls on the likelihood of the parent reporting that their child is in excellent health. The second model reports these relationships paired with and the effect of each discrimination over time, using Time 2 as the comparison period (Time 1 as the reference group).

Model 1 illustrated four statistically significant effects, in addition to the intercept. In terms of perceived discrimination, parental discrimination increased the likelihood of the PK reporting the child to be in excellent health (OR 1.13; CI 1.04–1.23). For every one-point increase on the parent discrimination variable, children with parents who had themselves experienced discrimination were 13% more likely to report being in excellent health. Given the mean and range of this variable, its effect size is relatively small. Perceived family discrimination had no statistically significant influence on child’s health. An inverse relationship was found for the relationship between perceived cultural discrimination and reporting excellent health (OR 0.94; CI 0.90–0.96). For every one-point increase on the cultural discrimination measure, children were 6% less likely to be in excellent health. Given the mean and minimum-maximum values for this variable, the effect size is potentially large.

In Model 1, a significant effect is shown over time concerning the child’s health. Children at Time 2 were more apt to be in excellent health than during Time 1, after controlling for all other variables in the model (OR 1.72; CI 1.33–2.23). Children were 72% more likely to report being in excellent health at Time 2. Only one of the other controls statistically influenced the child’s health. Parental university education increased the odds of a child being in excellent health (OR 1.62; 1.27–2.08). Children whose parents had a university education were 62% more likely to report being in excellent health. Child’s health was not significantly influenced by family income, child’s sex, or family’s year of arrival in Canada.

Model 2 includes three time-by-discrimination variables into the model. Unlike in Model 1, all three discrimination variables were found to influence the likelihood of reporting excellent health. Apart from the intercept, four main effects and one interaction effect were found to be statistically significant. Perceived discrimination experienced by both the parent (OR 1.19; CI 1.07–1.31) and family (OR 1.27; CI 1.03–1.56) worked to increase the chance of reporting excellent health. For every one-point increase in parent discrimination, children were 19% more likely to report being in excellent health. Similarly, for every one-point increase in family discrimination, children were 27% more likely to report being in excellent health. In both instances, after considering the each of the means and minimum-maximum values, this effect size is small. Similar to Model 1, cultural discrimination had a negative effect on the child’s parent-rated health (OR 0.88; CI 0.83–0.93). The effect size increased in Model 2, as children were 12% more likely to be in excellent health as cultural discrimination increased by one point. Considering the mean and minimum maximum value of this variable, this effect size is potentially quite sizeable.

Only one of the three interactions terms were statistically significant, indicating that only the effect of cultural discrimination changed over time. After controlling for all other variables in the model, cultural discrimination was found to have a different effect on children’s health in Time 2, compared to Time 1. As cultural discrimination increased over time, the likelihood of a child being in excellent health increased (OR 1.10; CI 1.03–1.18). This positive time-by-discrimination effect offsets the negative main effect that is associated with cultural discrimination, reducing the negative main effect previously reported. After summing the main (0.88) and interaction effects (1.10), we find an overall negative effect. In other words, compared to Time 1, parents in Time 2 are 2% less likely to report their children being in excellent health as the cultural discrimination variables increase by one point.

In Model 2, the effect of parent’s education maintained itself from Model 1 (OR 1.62; CI 1.27–2.08). Similar to Model 1, parent’s income, sex of the child, and length of time that the parent had spent in Canada did not influence the child’s health.

## Conclusions and Discussion

Our study is unique as it distinguishes the effects of various types of discrimination—parental, family, and cultural—measured over two times, on children’s health in six immigrant populations in British Columbia. It is also unique in that it not only focuses on children but also measures general health status, rather than illnesses.

Contrary to expectation, our results show that discrimination experienced by both the parent and family had a positive effect on the health of the child. Both parental and family discrimination are individual and direct, and possibly more tangible experiences compared to perceived discrimination of one’s cultural group. It is feasible that, as newcomers, family cohesiveness and social support are enhanced and work to buffer children’s health when racial discrimination is perceived. Further testing is needed to examine this notion, exploring the direct and indirect influences of racial discrimination on immigrant children’s health.

We found that perceived cultural discrimination, which we maintain as an indirect less tangible experience, had a meager negative effect on the health of the child. While this effect itself is small, when parents perceived high levels of cultural discrimination, the negative influence on the child’s health becomes substantial. This perhaps suggests that children (and their families) are able to more easily mitigate negative health consequences associated with low levels of culture-based discrimination; however, when cultural discrimination is extreme, the scales tip so to speak and this has a sizable negative affect children’s health. In saying this, the overall discrimination levels that were reported were quite low. Our results are particularly interesting since these low levels of discrimination had a decisive statistically significant effect on health.

Our results illustrate that racial discrimination has an influence on children’s health, though as we have illustrated the relationship between discrimination and health is complex. Perceived personal discrimination was less frequently reported than cultural-based discrimination. Over time, the former declined, while the latter increased. When examining the effect of discrimination on health over time, we found that the increase in cultural discrimination has a positive influence on health. This trend was also found when examining the general (main) effect of parent and family discrimination on health. We do not purport that discrimination has a positive effect on health; however, there could be a spurious artifact at work here. We suggest that discrimination may act as a catalyst that more tightly binds and bridges family and possibly ethnic community ties. Future work is needed to examine the growth of this social capital and its relationship with discrimination and health.

Limitations of our study relate to the sample and to our measures. First, the sample was not randomly selected, since it was not possible to do so; therefore, the generalizability of the results is limited. In addition, our sample includes families who speak the dominant language in six countries or regions. We did not include families who could not speak this language and consequentially this selection bias may lead to an overall sampling bias. In noting this, however, the large sample size across six immigrant populations will overcome much of the statistical limitations associated with sampling methods. Consequentially, while we found trends in the data, these findings should be read with caution.

Few studies of immigrant children populations examine general health rather than mental or emotional health outcomes. The UK Millennium Cohort Study found no statistically significant associations between measures of racism and risk of obesity (Kelly et al. 2013). In contrast, in the USA, experiences of Latina mother’s language-based discrimination was found to influence her child’s health, as measured by visits to the doctor for illness (Halim et al. 2013). Consistent with our findings, however, Halim et al. (2013) concluded that strong ethnic group attachment and a sense of belonging to one’s ethnic community may buffer the deleterious effects of racial discrimination. Priest et al. (2013) suggest that weaker associations between racial discrimination and physical health outcomes for children may reflect delayed onset of outcomes such as blood pressure, obesity, and other chronic illnesses indicating the need for more longitudinal studies.

Previous studies (Bécares et al. 2015; Paradies et al. 2015; Harris et al. 2015; Tran 2014; Priest et al. 2013; Kelly et al. 2013; Halim et al. 2013; Williams and Mohammed 2009; Pascoe and Smart Richman 2009; Williams et al. 2008; Miller and Kaiser 2001; Krieger 2000) show that racial discrimination has a negative effect influence on health outcomes, although mediators and moderators have been noted (Tran 2014; Halim et al. 2013; Pascoe and Smart Richman 2009; Noh and Kaspar 2003; Noh et al. 1999; Clark et al. 1999). One of the few longitudinal studies of immigrant populations and one of the few conducted outside the USA, the UK Millennium Cohort Study of mothers from five ethnic backgrounds, found direct association between experiences of maternal and family racism and children’s socioemotional development at age 11 years (Bécares et al. 2015). This occurred mainly because of the mother’s worsening mental health, and to some extent was related to increased harsh parenting practices.

Research has found that for some ethnic populations, social support (living in areas of ethnically dense concentrations) seemingly mediate the otherwise negative effect that discrimination (Jurcik et al. 2013; Das-Munshi et al. 2010). In other research we (George and Bassani 2015, George and Bassani 2013; George et al. 2012; Bassani and George 2012) found the importance of context when examining immigrant children’s health, which negated many US based research findings that associated poor health with ethnic neighborhood segregation. It is probable that the experiences of new immigrant groups differ from other more dominant ethnic groups in terms of their associations with health and racial discrimination. It appears that new immigrant populations may develop a sense of community and social support when treated racially, thereby shielding themselves from the otherwise negative effects of racial discrimination. In this regard, it may be that when parents and families experience discrimination protective mechanisms manifest.

Perceived discrimination is complex. More research across various countries and contexts is needed to understand the complex effects of racial discrimination on children’s health.

## APPENDIX: Items in Discrimination Measures

### Parent-Level Discrimination

Others discriminate against me.

I am treated as an alien by other Canadians.

I am reminded by others of my minority status.

### Family-Level Discrimination

Family member(s) have experienced:

- Unfairly fired or denied a promotion
- Not hired for a job for unfair reasons
- Unfairly treated by police
- Unfairly discouraged by teacher(s)
- Unfairly treated in buying\selling\renting a home
- Unfairly treated by neighbors

### Cultural Group-Level Discrimination

Canadians tend to look down on people from my country.

People from home country portrayed in media less fairly than other groups.

Landlords would rather rent an apartment to another group.

People from home country treated less fairly at government offices.

People from home country treated less fairly at stores.

People from home country treated less fairly when applying for jobs.

People from home country treated less fairly by police than people from other groups.
